# 肺低级别神经内分泌肿瘤的系统治疗

**DOI:** 10.3779/j.issn.1009-3419.2019.01.07

**Published:** 2019-01-20

**Authors:** 峥 王, 世钊 程, 方 周, 兴鹏 韩, 喜科 卢, 大强 孙, 逊 张

**Affiliations:** 300000 天津，天津市胸科医院胸外科 Department of Thoracic Surgery, Tianjin Chest Hospital, Tianjin 300000, China

**Keywords:** 神经内分泌肿瘤, 肺, 治疗, Neuroendocrine tumor, Lung, Therapy

## Abstract

肺部是神经内分泌肿瘤（neuroendocrine tumor, NET）的第二好发部位。肺类癌包括典型类癌和不典型类癌，属于低级别神经内分泌肿瘤。这一类肿瘤由于发病率较低，目前尚未得到广大医务工作者的重视。但有效的治疗不仅能提高患者的远期生存，还能控制患者症状，改善生活质量。本文分别阐述肺低级别神经内分泌肿瘤的流行病学和病理学特点、早期患者的治疗策略以及进展期患者的治疗策略。早期患者应当尽早进行手术治疗。进展期患者治疗方式包括化疗、SSAs、mTOR抑制剂、肽受体介导的放射性核素治疗、生物治疗以及靶向治疗。目前的研究结论大多来自其他部位的NETs研究外推而来，仍需针对肺低级别神经内分泌肿瘤患者进行特异性临床试验加以证实。

## Abstract (en)

The lung is the second most common site of neuroendocrine tumors (NETs). Typical and atypical carcinoids are low-grade NETs of the lung. These rare tumors have received little attention and education is needed for treating physicians. The article describes the classifcation of lung NETs, the epidemiology and pathological characteristics. When lung NETs are diagnosed at an early stage, surgical intervention is often curative. For advanced lung NETs patients, different treatment methods including chemotherapy, somatostatin analogs, m-TOR inhibition, peptide receptor radioligand therapy, and biologic systemic therapy are discussed. The conclusions are generally extrapolated from the outcome of extra-pulmonary carcinoids. Prospective randomized well-designed trials are urgently needed to inform current recommendations on systemic treatment.

神经内分泌肿瘤见于全身各个部位，是一类具有独特分子表型和临床特征的恶性肿瘤。大约30%的神经内分泌肿瘤（neuroendocrine tumor, NET）起源于肺，肺也是NETs的第二好发部位，仅次于胃肠道神经内分泌肿瘤^[1]^。发生于肺的神经内分泌肿瘤根据恶性程度排序分为低级别的典型类癌，然后到相对中级别的不典型类癌，再到高级别大细胞神经内分泌癌和肺小细胞癌^[2]^。尽管肺低级别神经内分泌肿瘤属于恶性肿瘤，但是其生物学行为相对惰性，通过积极地治疗依然可以获得长期生存甚至治愈疾病。

随着对肺低级别神经内分泌肿瘤这一疾病惰性生物学行为的认识逐渐增强以及疾病诊断准确性的提高，肺低级别神经内分泌肿瘤在人群中的发病率和患病率均有所增加。据权威数据统计，肺低级别神经内分泌肿瘤在美国和欧洲的发病率约0.2/10万-2/10万，男女比例大约8:1-10:1，占全部肺恶性肿瘤的1%-2%^[3-5]^。肺低级别神经内分肿瘤包括典型类癌和不典型类癌。典型类癌占全部胸部恶性肿瘤的1%，大约有10%的几率出现远处转移。不典型类癌占全部胸部恶性肿瘤的0.1%，大约有20%的几率出现远处转移^[6]^。除此之外，肺低级别神经内分泌肿瘤是幼儿及青少年最常见的肺部肿瘤^[7]^。70%-75%的肺低级别神经内分泌肿瘤为中心型的典型类癌，肿物直径为25 mm-34 mm^[8, 9]^。不典型类癌肿物直径更大，多为外周型且伴随着淋巴结的跳跃性转移^[10]^。最新研究结果^[11]^显示，不典型类癌患者的Ki67指数高于典型类癌，这也提示着不典型类癌具备更强的增殖能力。吸烟是肺低级别神经内分泌肿瘤的高危致病因素，尤其是不典型类癌^[12]^。由于肺低级别神经内分泌肿瘤多位于中心型且血供丰富，因此大多患者因咯血、咳嗽、哮喘或者反复的阻塞性肺炎而就诊。

典型类癌通常镜下肿瘤细胞形态比较温和、均一一致，核分裂像 < 2个/2 mm^2^，并且没有坏死。不典型类癌形态同样相对温和，但是核分裂像增长到2个/2 mm^2^-10个/2 mm^2^，同时偶见坏死。肺低级别神经内分泌肿瘤起源于Kulchitsky细胞，因此可以分泌生物活性多肽。但不同于胃肠道的神经内分泌肿瘤，肺低级别神经内分泌肿瘤很少出现库欣综合征、类癌综合征、肢端肥大症、脑炎、重症肌无力、高钙血症以及低血糖等副瘤综合征。尽管如此，由于肿瘤释放5-羟色胺入血进入左心而引发心脏疾病，肺低级别神经内分泌肿瘤的死亡率依然比较高^[10, 13]^。肺低级别神经内分泌肿瘤目前仍应用国际抗癌联盟（Union for International Cancer Control, UICC）公布的第7版TNM（tumor node）分期进行分期，但其分期的效果并不令人满意^[14]^，我们期待今后有更特异性的分期指南能够对肺低级别神经内分泌肿瘤进一步准确分期。

## 1 早期肺低级别神经内分泌肿瘤的手术治疗

早期肺低级别神经内分泌肿瘤的标准治疗方案是依然是手术治疗。典型类癌患者术后的5年、10年生存率均高达90%；而不典型类癌患者术后的5年生存率也可达到70%，10年生存率可达到50%^[15]^。截至目前，早期肺低级别神经内分泌肿瘤的标准治疗策略依然是手术治疗。研究结果显示，正电子发射计算机断层显像（positron emission tomography/computed tomography, PET/CT）在术前检查上并不能带来帮助，无论是典型类癌和不典型类癌的鉴别诊断还是纵隔淋巴结及其他远处转移的筛查^[16]^。近些年来，一些学者认为手术治疗需要同时进行系统性淋巴结清扫^[3]^。手术的方式包括肺叶切除术、袖式切除术以及楔形切除术。由于肺低级别神经内分泌肿瘤通常位于主支气管以及叶支气管，因此部分患者还需要接受隆凸成型术。王群教授等^[17]^近期纳入了美国监测、流行病学和最终结果（Surveillance, Epidemiology, and End Results, SEER）数据库7, 057例肺低级别神经内分泌肿瘤患者进行分析，结果显示手术治疗依然是治疗肺低级别神经内分泌肿瘤患者的基石，肺叶及亚肺叶切除患者的预后要优于全肺切除者。进一步分层分析结果显示，典型类癌患者亚肺叶切除术可以达到肺叶切除术的疗效，但是对于不典型类癌患者来说，肺叶切除术的疗效还是优于亚肺叶切除术。对于术前未经病理学证实的患者，术中可先行楔形切除术，后根据冰冻病理结果决定下一步治疗方案。如果术中冰冻证实其为典型类癌且切缘为阴性，术者还需对纵隔淋巴结进行冰冻采样。如果纵隔淋巴结术中冰冻为阴性则无需再进行肺叶切除术，反之，尽管病理学证实为典型类癌，但仍需进行肺叶切除术^[18]^。不典型类癌的治疗方案则需要参照非小细胞肺癌的治疗方案，需要进行肺叶切除术或者全肺切除术以及纵隔淋巴结清扫。如果术前高度怀疑为N2或者术前纵隔镜病理证实为N2的患者，需要通过多学科讨论来制定下一步治疗方案^[15]^。

局部病灶可切除但身体一般状态无法耐受根治性手术的患者可以行根治性或姑息性的射频消融术^[15]^。周围型病变无法耐受手术的患者可以行射频消融或放疗。对于中央型病灶的患者来说，我们可以考虑行内镜下切除、激光或者冷冻术，但是管腔外的病变比较广泛则需考虑行放疗或手术治疗。

肺低级别神经内分泌肿瘤术后同样需要长期随访。患者的局部复发较远处转移来说相对少见，局部复发可以考虑再次手术治疗或者一些其他的局部治疗。远处转移的患者需要联合局部治疗和全身治疗来控制疾病进展和减轻肿瘤负荷^[19, 20]^。

由于目前的数据资料十分有限，肺低级别神经内分泌肿瘤的术后辅助治疗仍然存在争议。典型类癌和不典型类癌单纯手术治疗的5年生存率分别达到90%-95%和60%-70%，因此目前大多数学者的观点认为接受根治性手术治疗且无淋巴结转移患者的下一步治疗则以临床密切随访为主^[5, 22, 23]^。

目前的指南主要建议对于姑息性切除以及残端阳性或者淋巴结转移的患者行进一步的术后辅助化疗或放疗^[22]^。美国国立综合癌症网络（National Comprehensive Cancer Network, NCCN）指南指出典型类癌的术后辅助化疗作用尚不明确，主要建议对Ⅱ期-Ⅲ期的不典型类癌进行术后辅助化疗^[23]^。欧洲神经内分泌肿瘤协会（European Neuroendocrine Tumor Society, ENETS）指南则建议对存在淋巴结转移的不典型类癌进行辅助化疗^[24]^。然而，目前的证据多为单中心的小样本量的回顾性研究，证据级别并不充分。

因此，术后辅助治疗能否带来生存上的获益尚无明确定论，还需要未来的前瞻性随机研究来加以证实。

## 2 进展期肺低级别神经内分泌肿瘤的治疗策略

来自美国SEER数据库的数据显示，12.9%的NETs患者在就诊的时候就发现了远处转移^[14]^。尽管肺低级别神经内分泌肿瘤占全部神经内分泌肿瘤的25%，但是关于进展期肺低级别神经内分泌肿瘤的系统治疗的研究仍然匮乏，目前的研究大都不是随机对照试验或者安慰剂对照试验。目前的研究多为小样本量单中心研究，其肿瘤的生物学行为及化疗的总有效率差异较大，因此并不能给我们带来明确的指示意义。很多的研究结论都是一些肺外肿瘤的研究结果外推而来。这些研究的治疗方式的设计并不足够严谨，并且混杂有一些目前已经弃用的治疗选择^[5, 19]^。此外，这些研究在治疗方式的选择上并未考虑到肺低级别神经内分泌肿瘤的惰性生物学活性。

进展期肺低级别神经内分泌肿瘤的治疗目的主要通过减轻患者症状和控制肿瘤生长来改善患者生活质量并延长患者的生存期^[25, 26]^。肿瘤的治疗需要顾及各个方面的综合情况，如就诊时间、肿物位置、肿瘤转移部位；患者的症状以及异位激素的分泌情况；局部治疗的并发症风险以及多学科讨论的意见。尽管无症状的高分化肺低级别神经内分泌肿瘤的生物学活性相对惰性，但是临床医生仍然需要视其为恶性肿瘤来进行密切随访。

### 2.1 常规化疗

同肺小细胞癌恰恰相反，肺低级别神经内分泌肿瘤患者的化疗结果令人失望，而典型类癌化疗的总缓解率（overall response rate, ORR）更低。尽管其无进展生存期（progression-free survival, PFS）和OS较不典型类癌更长，但是其原因更多的是由于其生物学行为的惰性^[27]^。经典的化疗药物如5-氟尿嘧啶、阿霉素、链脲霉素、达卡巴嗪、环磷酰胺以及替莫唑胺的客观有效率均小于30%，平均生存期约为42个月-120个月，3度-4度的化疗毒副反应大于10%^[19, 28]^。一项纳入204例NETs患者的研究，其中6.4%的患者为肺低级别神经内分泌肿瘤，该研究结果显示经典的依托泊苷+顺铂治疗客观有效率为23%，疾病控制率高达69%^[29]^。尽管肺低级别神经内分泌肿瘤患者对于化疗存在着一定的反应，但截至目前依然缺乏统一标准的治疗方案。

### 2.2 SSAs治疗典型类癌

具有神经内分泌功能的肺低级别神经内分泌肿瘤患者需要特殊的对症治疗来改善相应的症状，如腹泻、面色潮红、腹痛、低血压以及血管痉挛。除此之外，研究结果显示该类治疗还可以起到控制疾病进展的作用^[30]^。在典型类癌患者中，生长激素抑制素受体常常呈现过表达^[27]^。SSAs（somatostatin analogs）作用于生长激素抑制素受体，阻断其释放多肽类和有机胺的释放，因此来减轻患者的症状。目前应用于临床治疗的药物有两种，分别为奥曲肽和兰瑞肽。这两种药物毒副反应均较轻，最为常见的不良反应为胆囊结石，因此建议患者每6个月复查腹部彩超。

类癌危象相对罕见，通常是由于大量的有机胺释放入血所致，导致患者出现低血压和面色潮红。这一现象常常出现在手术过程中或者全麻过程中，因此外科医生需要术前进行SSAs药物预处理。

SSAs不仅能改善患者的症状，还能抑制肿瘤的增殖。研究结果显示，在胰腺癌和胃肠道肿瘤中，无论其是否存在神经内分泌功能，SSAs均能带来生存上的改善^[30, 31]^。截至目前，唯一的一项纳入了肺和胸腺NETs的LUNA研究结果显示，帕瑞肽、依维莫司或者二者联合应用的治疗在第9个月的PFS上均取得有效性^[32]^，分别为39%、33.3%以及58.5%。因此，LUNA研究证实SSAs对于伴有神经内分泌功能的肺低级别神经内分泌肿瘤患者来说是一项可行的治疗选择。但一些学者认为这类药物只是适用于治疗有症状的患者。

### 2.3 mTOR抑制剂

随着研究证实肺低级别神经内分泌肿瘤患者呈现出雷帕霉素靶蛋白通路活性的增加，依维莫司作为作为一种mTOR抑制剂在肺低级别神经内分泌肿瘤治疗中存在着巨大潜能。IGF-1通路的上调会导致依维莫司的耐药，而奥曲肽则会使得IGF-1通路下调。一项Ⅲ期临床试验RADIANT-2研究结果显示，依维莫司+长效奥曲肽同单纯长效奥曲肽在治疗类癌综合征的患者上PFS上存在优势，二者分别为16.4个月和11.3个月（*P*=0.026）^[33]^。在肺低级别神经内分泌肿瘤患者（*n*=44）的亚组分析中，PFS同样获得了统计学上的趋势，两组患者分别为13.6个月和5.6个月（*P*=0.228）。由于该项研究入组患者偏少，同时纳入了全身各个部位的NETs患者且均为功能性神经内分泌肿瘤，因此对于依维莫司在治疗全部患者的证据并不充分。不良事件主要为1级-2级。3级-4级的毒副反应主要是粘膜炎、皮疹以及腹泻。

随后的RADIANT-4研究则针对非功能性肺部和消化道神经内分泌肿瘤进行了研究，两组患者分别接受依维莫司和安慰剂治疗，结果显示二者的中位PFS存在显著差异，分别为11个月和3.9个月（*P* < 0.01）^[34]^。分层分析显示无论是肺低级别神经内分泌肿瘤还是消化道或者原发部位未知的神经内分泌肿瘤患者均能得到生存上的获益。

因此，RADIANT-2研究证实长效奥曲肽联用依维莫司同单药长效奥曲肽相比，不但安全还能改善功能性神经内分泌肿瘤患者的生存。而RADIANT-4研究则针对的是非功能性肿瘤患者进行研究，因此目前认为依维莫司仅仅适合在非功能性肺神经内分泌肿瘤患者进行单药治疗。

### 2.4 生物治疗和抗血管生成药物

NETs往往血供丰富并且呈现促血管生成基因（*VEGF*, *VEGFR*, *PDGF*, *PDGFR*, *IGF-1*, *IGFR*）的过表达^[35]^。因此，抗血管生成药物无论是口服还是静脉给药方式均已被推荐应用于NETs的治疗。一项纳入了29例进展期NETs（52%为肺低级别神经内分泌肿瘤）的研究应用替莫唑胺/沙利度胺方案治疗，结果显示ORR为25%，疾病进展时间（time to progression, TTP）为13.5个月^[36]^。舒尼替尼是一类VEGFR-1-3和PDGFR-A-B的酪氨酸激酶抑制剂。一项研究纳入了107例类癌患者应用舒尼替尼进行治疗，结果显示加用舒尼替尼组在胰腺神经内分泌肿瘤和类癌组中治疗结果分别为ORR（16.7% *vs* 2.4%），DCR（68% *vs* 83%），TTP（7.7个月*vs* 10.2个月），1年生存率（81.1% *vs* 83.4%）^[37]^。在PAZONET研究中，44例进展期NETs患者中（9.5%为肺低级别神经内分泌肿瘤）帕唑帕尼治疗效果显示ORR为9.1%，DCR为56.8%，PFS为9.5个月^[37]^。一项Ⅱ期临床试验纳入了44例接受奥曲肽治疗的进展期类癌（9.1%为肺低级别神经内分泌肿瘤），两组分别接受贝伐单抗和聚乙二醇化干扰素诱导治疗，而后继续使用该药物直至肿瘤进展。结果显示贝伐单抗组效果优于聚乙二醇化干扰素组，ORR和DCR分别为（18% *vs* 0%）和（95% *vs* 68%）^[38]^。而另一项研究应用贝伐单抗/甲氧基雌二醇治疗31例进展期类癌（13%肺低级别神经内分泌肿瘤），结果令人十分振奋，ORR达到68%，PFS为11.3个月^[39]^。

α-干扰素是机体免疫细胞产生的一种细胞因子，具有抗血管生成的活性，是治疗NETs的经典药物。一项纳入了27例接受干扰素治疗的进展期肺低级别神经内分泌肿瘤的研究结果显示其疾病控制率（disease control rate, DCR）为14.8%，而且奥曲肽联合干扰素可以延长类癌姑息性切除术患者的TTP^[40]^。SWOG-0518试验纳入了402例进展期NETs，比较了长效奥曲肽/贝伐单抗和长效奥曲肽/干扰素的疗效差异，结果显示贝伐单抗组的TTP优于干扰素组（5.个月*vs* 9.9个月，*P*=0.003），而两组患者的PFS则无显著差异（16.6个月*vs* 15.4个月，*P*=0.55）^[41]^。一项*meta*分析纳入了414例接受干扰素治疗的NETs，纳入研究报道ORR为12%-20%，DCR为32%-53%，生化应答率39%-49%，结果证实了患者接受干扰素的维持治疗可以带来生存上的获益^[5]^。

生物治疗包括抗血管生成药物在内在治疗神经内分泌肿瘤中存在的一定的疗效。尽管在肺低级别神经内分泌肿瘤中缺乏高质量的证据支持，但是在一些指南中还是将该类药物纳入其中。

### 2.5 靶向药物治疗

尽管在部分肺低级别神经内分泌肿瘤患者中会出现c-kit、PDGFR-A-B以及EGFR的表达，但是仍然没有足够的数据证实肺低级别神经内分泌肿瘤患者可以从伊马替尼或厄洛替尼的治疗中获益^[42]^。但是，生长激素类似物是一类作用于生长抑素受体的药物，该药物被证实可以控制大约70%NETs的生长激素过分泌所带来的不适症状。短效的SSAs通常用来控制过敏反应以及控制类癌综合征直至长效的药物能够达到稳态的水平。

特罗司他乙酯是一类色氨酸羟化酶抑制剂，美国FDA已批准了特罗司他乙酯的新药申请，它是首个被批准用于类癌综合症的口服治疗药物^[43]^。既往的传统药物如酮康唑、美替拉酮、依托咪酯以及米非司酮也同样可以治疗库欣综合征^[44]^。如果系统治疗仍不能改善内分泌症状，可以考虑一些姑息治疗的手段如姑息性肝切除术、灌注化疗、射频消融以及双侧肾上腺切除术。

### 2.6 肽受体介导的放射性核素治疗

有研究显示肽受体介导的放射性核素治疗（peptide receptor radionuclide therapy, PRRT）对于晚期高表达肽受体放射性核素治疗的NETs治疗有效^[45]^。PRRT不仅能够改善患者症状，还能明显改善患者的生存质量，但是缺乏针对肺低级别神经内分肿瘤的前瞻性临床Ⅲ期证据。PRRT的出现无疑为国内神经内分泌瘤患者带来希望，然而由于这种技术及实施前的检查专业性较强，以及国内对这种肿瘤的认识起步较晚，一直没有开展PRRT的治疗。2018年2月起，北京大学肿瘤医院将在国内率先开展基于Lu-177的PRRT前瞻性临床研究，目前已经开始招募患者，我们期待该项目的研究结果公布，并希望该项目能够早日应用于我国的NETs患者。此外，将PRRT与其他联合应用，例如化疗、生物治疗以及靶向治疗，也可作为晚期进展期肺低级别神经内分肿瘤的潜在治疗方法。

### 2.7 随访策略

肺低级别神经内分泌肿瘤患者同样需要进行常规的术后随访。典型类癌患者术后3个月和6个月进行影像学检查，随后每年进行复查即可。不典型类癌患者则需要进行更为严格的术后监测，术后3个月和6个月进行复查后，仍需要每6个月进行常规复查^[5]^。对于进展期肺低级别神经内分泌肿瘤患者来说，其随访策略目前尚无统一标准^[46]^。随访策略个体化差异较大，主要根据其初治的基线特征、新发症状以及初治的治疗方案制定。CgA可以用来监测疾病进展，但是其应用频率及应用持续时间尚不清晰。未来需要更为详细的指南来指导患者的随访策略。

## 3 结论

肺低级别神经内分泌肿瘤是一类相对少见的惰性生物学行为的恶性肿瘤，但是根据病理分期及生物学行为的不同，仍然会有大约5%-25%的患者死亡。早期患者应当尽早进行手术治疗。进展期患者治疗方式包括化疗、SSAs、mTOR抑制剂、肽受体介导的放射性核素治疗、生物治疗以及靶向治疗。通过测序技术检测肿瘤驱动基因有助于进一步筛选有效的治疗靶点，同样在未来我们相信液体活检一样能在肺低级别神经内分泌肿瘤上得到应用。近年来的研究热点免疫治疗抗PD-1/PD-L1和抗CTLA4等在NETs患者的安全性和有效性的临床转化研究正在开展，我们也期待未来免疫治疗可以成为肺低级别神经内分泌肿瘤的治疗方法。

遗憾的是，由于对这类疾病认识不足，各个中心的治疗策略尚不完全一致，缺乏统一的标准。与此同时，目前对肺低级别神经内分泌肿瘤的治疗方法很多都是根据其他部位的NETs的治疗数据推测而来，针对肺低级别神经内分泌肿瘤患者的临床试验尚不完善，因此我们仍期待设计精良的大型随机对照临床试验的数据来指导未来的治疗。
